# Angle Class III malocclusion with anteroposterior and vertical discrepancy in the final stage of growth

**DOI:** 10.1590/2177-6709.22.3.109-118.bbo

**Published:** 2017

**Authors:** Marcelo B. P de Arruda

**Affiliations:** 1 Universidade Federal de Mato Grosso do Sul, Orthodontics Department, (Campo Grande/MS, Brazil). Diplomate, Brazilian Board of Orthodontics and Facial Orthopedics.

**Keywords:** Angle Class III malocclusion, Open bite, Maxilla.

## Abstract

Angle Class III malocclusion is characterized by an anteroposterior dental discrepancy with or without anteroposterior and vertical skeletal changes. Patients usually seek orthodontic treatment because facial appearance is compromised in most cases. The present study describes the clinical case of a 12-year and 6-month-old girl in her final stage of pubertal growth presenting Class III malocclusion with anteroposterior and vertical discrepancies. Initial treatment consisted of maxillary expansion using a Hass expander followed by the use of a Petit facemask for a minimum of 16 hours a day. During corrective treatment, Class III elastics were used to complement protraction. At the end of the treatment, skeletal discrepancy had improved, and the ANB angle increased from 0 to 2^o^. Angle Class III malocclusion, anterior crossbite and open bite were corrected. This case was presented to the Committee of the Brazilian Board of Orthodontics and Facial Orthopedics (BBO) as part of the requisites to become a BBO Diplomate.

## RESUMO

A má oclusão de Classe III de Angle tem como característica uma discrepância dentária anteroposterior, que pode ou não estar acompanhada por alterações esqueléticas tanto no sentido anteroposterior quanto no vertical. O aspecto facial fica comprometido na maioria dos casos, levando o paciente a procurar o tratamento ortodôntico. O presente artigo descreve o caso clínico de uma paciente com doze anos e seis meses de idade, portadora de má oclusão de Classe III, com discrepância anteroposterior e vertical, em fase final de crescimento. O tratamento inicial consistiu de expansão maxilar com o disjuntor de Haas e utilização da máscara facial de Petit por no mínimo 16 horas/dia. Na fase de tratamento corretivo, foram usados elásticos Classe III como complemento à tração reversa. Ao término do tratamento, obteve-se melhora na desarmonia esquelética, com aumento do ângulo ANB de 0^o^ para 2^o^. Quanto ao padrão dentário, corrigiu-se a relação de Classe III de Angle, o leve cruzamento entre os incisivos e a mordida aberta anterior. Esse caso foi apresentado à Diretoria do Board Brasileiro de Ortodontia e Ortopedia Facial (BBO) como parte dos requisitos para a obtenção do título de Diplomado pelo BBO.

## INTRODUCTION

**Figure 1:**
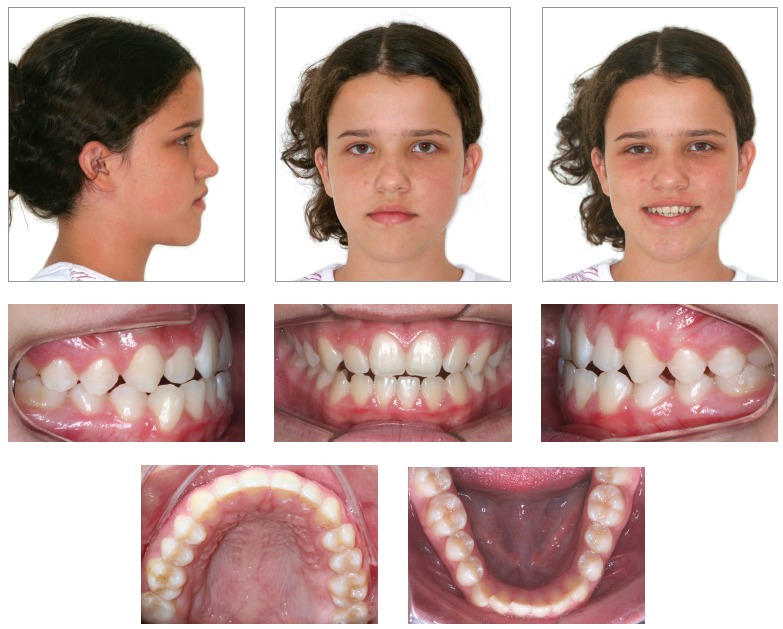
Baseline facial and intraoral photographs.

A healthy 12-year and 6-month-old girl presented for an initial clinical examination. Her main complaint was that her mandibular teeth were in an anterior position in relation to her maxillary teeth, which compromised her facial esthetics (Fig 1). According to her mother, the girl had undergone previous dental examinations, and all the other dentists had indicated surgery. All the patient’s teeth were intact, but she had anterior crossbite and open bite and discrete tongue thrust. According to the mother, malocclusion had a hereditary component of paternal origin.

## DIAGNOSIS

The patient had both vertical and anteroposterior skeletal discrepancies, with an ANB angle of zero degrees, overgrowth of the mandible and a vertical growth pattern (SN.GoGn = 43^o^, FMA = 28^o^). Both the maxilla, more markedly, and the mandible were retruded in relation to the cranial base, which resulted in a concave profile (SNA = 77^o^ and SNB = 77^o^, convexity angle = -2^o^) (Figs 1 and 4). A wrist and hand radiograph revealed that the patient was at the end of her pubertal growth spurt, and her bone age was close to 14 years, which may have led other dentists to indicate orthognathic surgery (Fig 3).

**Figure 2:**
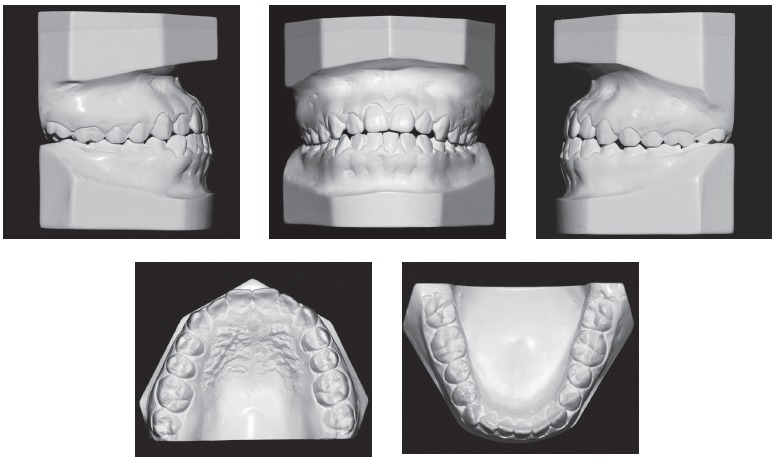
Baseline casts.

**Figure 3:**
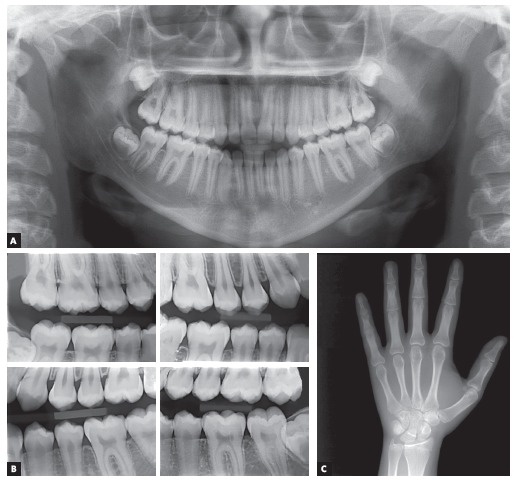
Baseline panoramic (A), interproximal (B) and hand and wrist (C) radiographs.

**Figure 4:**
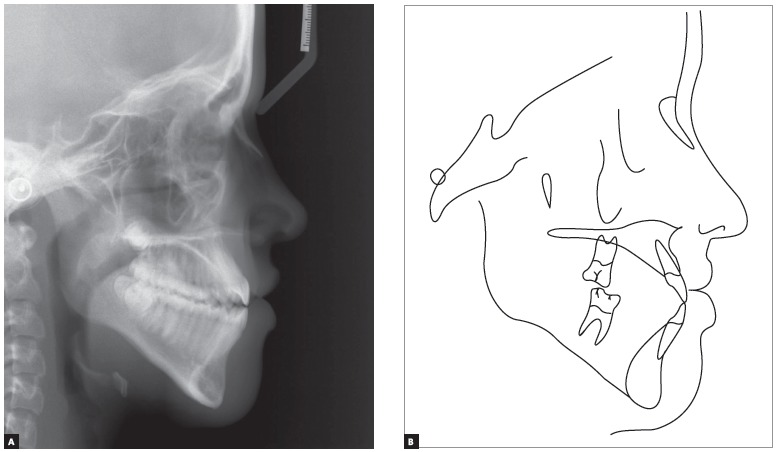
Baseline cephalometric profile radiograph (A) and cephalometric tracing (B).

Dental examination (Fig 2) revealed that the patient had Angle Class III malocclusion, maxillary and mandibular incisor protrusion (1-NA = 7 mm and 1-NB = 8 mm), mandibular midline deviation of 2.5 mm to the left, negative overjet and overbite, both of -2 mm, mandibular anterior crowding of -2 mm and anterior crossbite. The patient had no CO-CR deviation.

The analysis of panoramic and interproximal radiographs (Fig 3) confirmed that she had all teeth, including third molars, and no abnormalities that might compromise orthodontic treatment.

She had a straight profile and labial sealing associated with anteroposterior middle third deficiency and a long lower third of the face (Figs 1 and 4).

The following results were defined as the objectives of orthodontic treatment: to harmonize profile by improving skeletal positioning, to correct negative mandibular discrepancy and to retract mandibular incisors; to expand the maxilla to ensure a more effective protraction and to maintain facial height and prevent its increase. The treatment also included the restoration of ANB balance and the correction of the Class III relation. As the patient’s mandibular growth was limited, the plan included the preservation of cranial base positioning to avoid increasing the mandibular plane angle. For that purpose, the fundamental role of cooperation - particularly in the use of Class III elastics and the Petit facemask for protraction - was emphasized for the patient and her family. Midline correction was also planned. Finally, treatment was expected to significantly improve esthetics, as well as dental and skeletal patterns.

## TREATMENT PLAN

A two-phase treatment plan was prepared. The first phase consisted of maxillary expansion using a Hass expander, followed by the use of the Petit facemask for a minimum of 16 hours a day. In the second phase, Class III elastics would be used as a complement to protraction; in case the response was not positive, orthognathic surgery might still be used as an alternative treatment. 

## TREATMENT PROGRESSION

Initially, a Haas expander was placed in the maxillary arch for rapid palate expansion for 21 days. After that, the protraction facemask was connected to the same Haas expander, with the center of resistance placed at the canines, and application of a force of 350 g. The patient should wear the facemask for at least 16 hours a day. A fixed standard Edgewise appliance was placed in the maxilla (slot = 0.022 x 0.028-in), and 0.014-in, 0.016-in, 0.018-in and 0.020-in wires were used for alignment and leveling.

Six months later, the Haas expander was removed, and fixed appliance placement was completed: double tubes were bonded to the maxillary first molars and single tubes, to the maxillary second molars. Alignment and leveling were achieved using 0.018-in and 0.020-in wires with e-loops between the maxillary lateral incisors and the canines, to which the facemask elastics were connected. The patient wore the facemask only at night, for an average of 10 to 12 hours a day.

Eleven months later, the use of the facemask was discontinued, and control with Class III elastics started. A space-closing archwire was produced using a bull loop. For completion, a rectangular 0.019 x 0.026-in archwire was placed, and Class III (1/4-in light) elastics were used for 12 hours to improve intercuspation, in addition to vertical elastics (1/8-in heavy) placed in the premolar region, which should be used during sleep. After the fixed appliance was removed, a wraparound retainer was adapted to the mouth.

Six months after the beginning of the treatment, a fixed standard Edgewise (0.022 x 0.028-in) appliance was placed in the mandibular arch. Simple tubes were bonded to the first molars and lower tubes, to the second molars, and alignment and leveling were achieved with stainless steel 0.014-in, 0.016-in, 0.018-in and 0.020-in wires and a space-closing archwire with a 0.019 x 0.025-in bull loop. For completion, a rectangular 0.019 x 0.026-in archwire was used. Class III (¼-in light) elastics were used for 12 hours to improve intercuspation, and vertical elastics (1/8-in heavy) in the premolar region were used during sleep. After the fixed appliance was removed, a 3 x 3 retainer made with 0.7-mm wire was bonded.

## TREATMENT RESULTS

The examination of final records revealed that the initially planned objectives were achieved. In the maxilla, anterior protraction was achieved with the use of the Petit facemask, which improved SNA angle from 77 to 80^o^, although the patient had already reached the final stage of her pubertal growth spurt. The pattern remained vertical because the ‘y’ axis remained at 60^o^ from the beginning to the end of the treatment. Maxillary intermolar distance, 56 mm from the beginning to the end of the treatment, was preserved despite initial palate expansion. The analysis of dental pattern revealed that the 1.NA angle improved, as there was a reduction from 29 to 22^o^ and changes in the linear positioning of incisors (1-NA), which went from 7 mm to 8 mm (Table 1). The changes were beneficial and improved the anteroposterior maxilla-mandible relation, as well as the inter-relation between maxillary and mandibular incisors.

**Table 1:** Baseline (A) and final (B) cephalometric values.

	Measurements		Normal	A	B	A/B Diff.
Skeletal pattern	SNA	(Steiner)	82^o^	77^o^	80^o^	3
	SNB	(Steiner)	80^o^	77^o^	78^o^	1
	ANB	(Steiner)	2^o^	0^o^	2^o^	2
	Wits	(Jacobson)	♀ 0 ±2 mm ♂ 1 ±2 mm	0mm	2mm	2
	Angle of convexity	(Downs)	0^o^	-2^o^	4^o^	6
	Y-axis	(Downs)	59^o^	60^o^	60^o^	0
	Facial angle	(Downs)	87^o^	89^o^	88^o^	1
	SN-GoGn	(Steiner)	32^o^	43^o^	42^o^	1
	FMA	(Tweed)	25^o^	28^o^	30^o^	2
Dental pattern	IMPA	(Tweed)	90^o^	87^o^	88^o^	1
	1.NA (degrees)	(Steiner)	22^o^	29°	22^o^	7
	1-NA (mm)	(Steiner)	4 mm	7	8	1
	1.NB (degrees)	(Steiner)	25^o^	25^o^	28^o^	3
	1-NB (mm)	(Steiner)	4 mm	8mm	6mm	2
	- Interincisal angle	(Downs)	130^o^	126^o^	128°	2
	1-APo	(Ricketts)	1 mm	6mm	6mm	0
Profile	Upper lip - S-line	(Steiner)	0 mm	-2mm	2mm	4
	Lower lip - S-line	(Steiner)	0 mm	1mm	4mm	3

**Figure 5:**
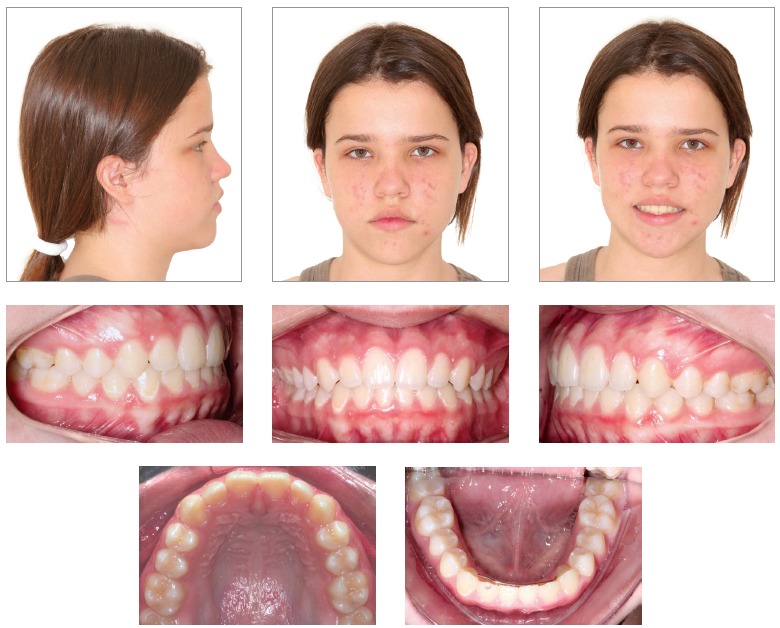
Final facial and intraoral photographs.

**Figure 6:**
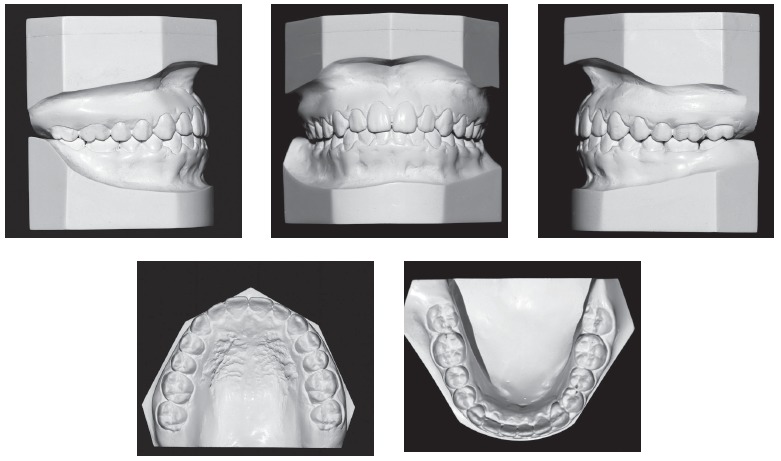
Final casts.

**Figure 7:**
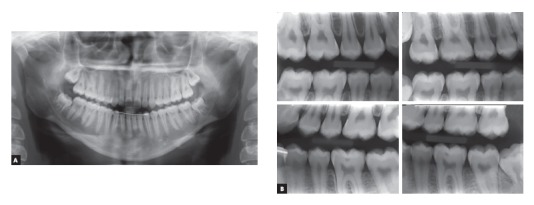
Final panoramic (A) and interproximal (B) radiographs.

In the mandible, there was a discrete change in the anteroposterior position of the cranial base, with a slight anterior movement followed by backward movement of incisors. A decrease of 1-NB from 8 mm to 6 mm was probably a result of the use of Class III elastics during treatment. This also led to a discrete improvement of skeletal discrepancy, with an increase of the ANB angle from 0 to 2^o^ (Table 1). Angle Class III malocclusion, crossbite between incisors and anterior open bite were corrected (Figs 5 and 6). Figure 7 shows the successfully achieved parallel position of the roots. A recommendation was made for extraction of third molars.

**Figure 8:**
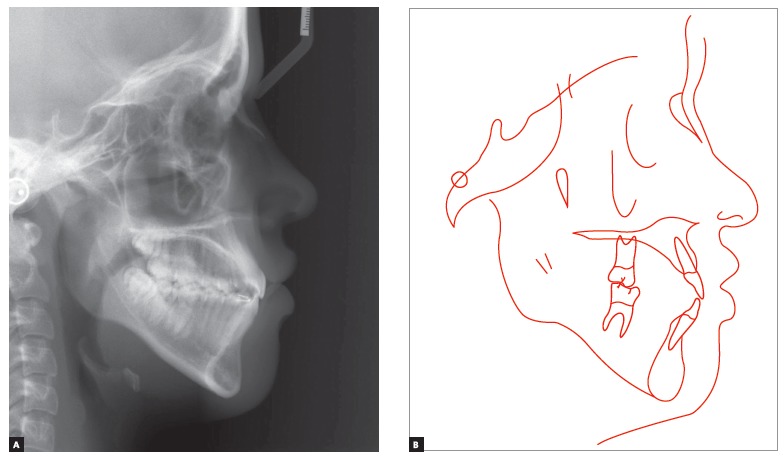
Final cephalometric profile radiograph (A) and cephalometric tracing (B).

Patient esthetics improved because of the position of the upper lip. Changes resulted in a slightly convex profile, as convexity angle went from -2 mm to 2 mm (Figs 5 and 8).

## FINAL CONSIDERATIONS

Angle Class III malocclusion, based on an anteroposterior dental relation, is more serious when associated with skeletal discrepancies resulting from maxillary deficiency, mandibular excess, or a combination of both. These changes may compromise facial profile. Treatment planning requires the use of lateral radiographs and other routine radiographic studies, as well as the evaluation of dental characteristics using clinical examination and analysis of diagnostic casts. The analysis of genetic factors should take into consideration not only facial characteristics - such as cephalometric characteristics of parents, siblings and other relatives -, but also information about possible previous interventions that other members of the family might have undergone^2^. 

Treatment options to correct this anomaly involve several factors. When the patient has not reached pubertal growth spurt, an early intervention is indicated, with the use of a facemask for maxillary protraction, usually together with palatal expansion.^3^ ^-^ ^7^

Palatal expansion^8^ is essential when a facemask is used, as it favors the achievement of a more anterior placement of the maxilla and improves the relation with the mandible, resulting in satisfactory occlusion. However, patient collaboration is fundamental^9^ ^,^ ^10^ ^,^ ^11^. In the case here reported, the patient was informed about the benefits of a treatment for which surgery would be a last resource. For that purpose, the use of reverse pull and Class III elastics was the first option. It was readily adopted and elicited excellent patient cooperation. To stimulate cooperation, the patient was told that the facemask would substantially improve her esthetic and facial profile.

**Figure 9:**
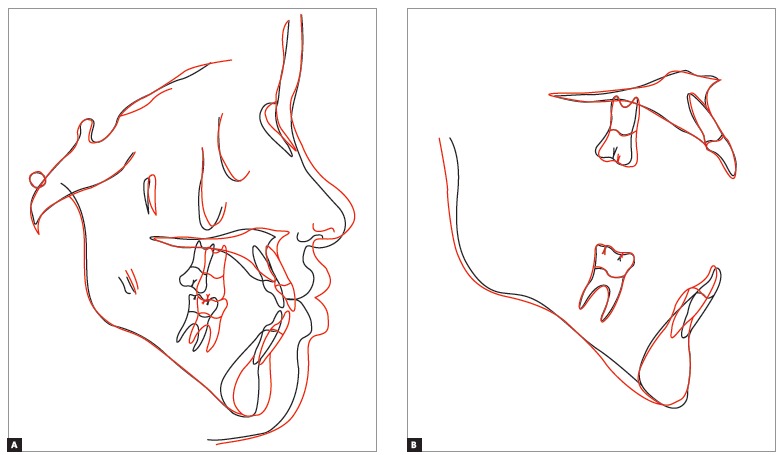
Total (A) and partial (B) superimpositions of baseline (black) and final (red) cephalometric tracings.

The analysis of dental characteristics revealed that the patient had Class III molar and canine relations, and that these relations were more marked in the left side. At the end of the treatment, the patient was advised to get her third molars extracted in the future. The records obtained at treatment completion (Figs 7 and 8) and the superimpositions of initial, final and control cephalometric tracings (Fig. 9) revealed that the result achieved after the removal of the orthodontic appliance was satisfactory, which illustrates the efficiency of the treatment that was planned and executed.
